# The male of *Megacormus granosus* (Gervais, 1844) with comments on its hemispermatophore (Scorpiones, Euscorpiidae)

**DOI:** 10.3897/zookeys.504.9027

**Published:** 2015-05-18

**Authors:** Edmundo González-Santillán, Fernando Alvarez-Padilla

**Affiliations:** 1 Laboratorio de Aracnología, Departamento de Biología Comparada, Facultad de Ciencias, Universidad Nacional Autónoma de México. Av. Universidad 3000, Circuito Exterior S/N, Delegación Coyoacán, C.P. 04510 Ciudad Universitaria, D.F. México

**Keywords:** Euscorpiidae, Orizaba, Veracruz, Mexico

## Abstract

The male of *Megacormus granosus* is described for the first time and the female redescribed. A homology scheme proposed recently is applied to hemispermatophore structures. The specimens were collected in an oak forest from Pico de Orizaba Volcano at an average altitude of 2340 m. All adult males were collected by pitfall traps, whereas all adult females and both sex immatures were collected using Berlese funnels, suggesting that males are comparatively more mobile within the leaf litter layer, probably due to mating season.

## Introduction

The Mexican genus *Megacormus* (Karsh, 1881) is comprised of four species, *Megacormus granosus* (Gervais, 1844); *Megacormus segmentatus* Pocock, 1900; *Megacormus gertschi* Díaz Nájera, 1966 and *Megacormus grubbsi* Sissom, 1994. All species of *Megacormus* are restricted to the slopes of the Sierra Madre Oriental and the costal lowlands of the Gulf of Mexico, ranging in altitude from 300 m to over 2300 m. They prefer habitats with high relative humidity such as oak, oak-pine and evergreen tropical forest, and are found in these communities within the leaf litter, outcrop crevices, decaying logs or under rocks. Their distribution includes the states of Hidalgo, Oaxaca, Puebla, Querétaro, San Luis Potosí, Tamaulipas, and Veracruz (Sissom 2000).

**Figures 1–6.**
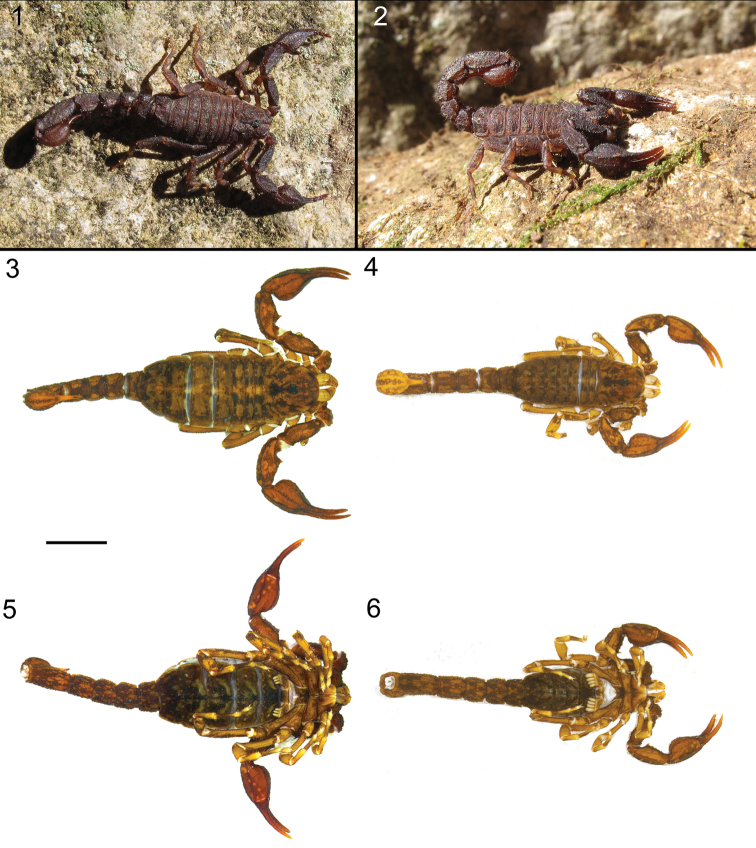
*Megacormus granosus* (Gervais, 1844). **1** ♂ habitus alive **2** ♂ habitus alive **3, 4** habitus, dorsal ♀ and ♂ **5, 6** habitus ventral ♀ and ♂. Scale bar 2.0 mm.

*Megacormus granosus* was originally described as a species of *Scorpio* Linnaeus, 1758 from a single female (Soleglad 1976), but its description and illustrations became obsolete as more scorpion diversity was discovered. The only character used to define this species was the dense granulation on the cuticular dorsal surfaces (Figures 1, 2). The second described species of the genus was *Megacormus segmentatus* Pocock, 1900, distinguished by the presence of a distinct furrow between the pectinal marginal and median lamella, which is absent in *Megacormus granosus*. Subsequently *Megacormus segmentatus* was considered as a subspecies of *Megacormus granosus* by Hoffmann (1931). The third species, *Megacormus gertschi*, was diagnosed by having a greater number of trichobothria on the patella prolateral surface and the presence of scalloping on pedipalp chela fingers. The first comprehensive revision of these taxa was done by Soleglad (1976), who recognized three species by reverting *Megacormus granosus segmentatus* status to its original designation as *Megacormus segmentatus*. Sissom (1994) added a fourth species and illustrated for the first time the hemispermatophores of *Megacormus grubbsi* and *Megacormus segmentatus*, but to date the hemispermatophore of *Megacormus granosus* has never been documented. Although Sissom’s (1994) extended descriptions, diagnoses and key to the species of *Megacormus* are in use, variation of diagnostic characters, *i.e.* trichobothrial counts, pectinal counts, etc., are not documented appropriately due to the paucity of specimens in scientific collections, except perhaps for *Megacormus gertschi* (Sissom 1994) and in this contribution *Megacormus granosus*.

The hemispermatophore constitutes one of the most informative character systems in suprageneric phylogenetic studies, at least in Bothriuridae Simon, 1880 and Vaejovidae Thorell, 1876 (Mattoni et al. 2011, González-Santillán and Prendini 2014); it has shown little value in diagnosing species of Euscorpiidae (Jacob et al. 2004) or Diplocentridae (Santibañez-López and Francke 2013) however. These results are congruent with the prediction of Song and Bucheli (2010) and the hypothesis of Peretti (2010), that the complex interaction of genitalic functional units is informative at several levels of the phylogeny and taxonomic hierarchy, but structures in related species that perform the same function tend to have the same selective pressure, thus presenting little variation. The hemispermatophore of the genus *Megacormus* is putatively informative (Sissom 1994: p. 269, figures 8–10), but its microstructure has never been studied in detail.

The unsatisfactory working terminology and concomitant absence of homologies in the hemispermatophore of the Order Scorpiones have promoted a plethora of nomenclatures, hindering the correct interpretation of homologies (Lamoral 1979, Stockwell 1989). Recently, González-Santillán and Prendini (2013, 2014) consolidated a terminology used by previous authors (Stockwell 1989, Sissom 1994), upon which they established a homology scheme for the hemispermatophore of the subfamily Syntropinae (Vaejovidae). In this contribution we extend that terminology and homologies to the scorpion family Euscorpiidae Laurie, 1896, when possible, applying it to the hemispermatophore of *Megacormus granosus*.

## Methods

All scorpions were collected by sifting leaf litter processed with Berlese funnels and pitfall traps. Specimens are deposited in the Laboratorio de Aracnología de la Facultad de Ciencias, Universidad Nacional Autónoma de México (UNAM), Mexico. Measurements in mm were taken with an ocular micrometer. Illustrations were produced with a Nikon SMZ 1000 dissecting stereomicroscope and a Nikon Eclipse E200 with a camera lucida. Photomicrographs were taken with a digital camera Nikon DS-U3 under LED illumination and Ultraviolet (UV) light using a 20W Techno Lite® bulb mounted in a desktop stand lamp. Hemispermatophores were digested with pancreatine (Alvarez-Padilla and Hormiga 2007), cleared with clove oil and mounted in temporary slides for illustration (Coddington 1983).

Nomenclature follows Stahnke (1970) except for pedipalp and metasomal carination, leg setation, spinules and the hemispermatophore, which follows González-Santillán and Prendini (2013), and some capsular terminology used by Jacob et al. (2004) for the genus *Euscorpius*, the putative sister group of *Megacormus*. Pedipalp chela dentition is modified from Soleglad and Sissom (2001) by recognizing six denticle types: retrolateral (Rl), retrolateral accessory (Reac), median (Me), median accessory (Meac), prolateral (Pl) and prolateral accessory (Prac). Prolateral and retrolateral translucent macrosetae delimit 10 positions denoted by roman numerals (Position I–X, Figures 12, 13), where a set or subset of those denticles are localized, following the illustration in González-Santillán and Prendini (2013, fig. 12). Hemispermatophore abbreviations are as follow: cap, capsule; c-lp, crown-like process; dl, distal lamina; dsp, dorsal spiculate process; dt, dorsal trough; t, trunk; tf, truncal flexure; vsp, ventral spiculate process; vt, ventral trough.

### Locality description

Specimens were collected in a 15–20-year old oak forest near the boundary of the Pico de Orizaba National Park ca. two kilometers southwest from Atotonilco de Calcahualco, Veracruz. Two plots, of one hectare each, were established with the following central coordinates. Plot I 19°8'17.4"N, 97°12'16.2"W, altitude 2,300 m. Plot II 19°8'30.2"N, 97°12'21.5"W, altitude 2388 m. Three expeditions were conducted, two in May 21–30^ th^ and October 4–14^th^ 2012 and the third in February 15–24^th^ 2013.

## Results

### Systematics Family Euscorpiidae Laurie, 1896 Genus *Megacormus* Karsch, 1881

#### Megacormus granosus

Taxon classificationAnimaliaScorpionesEuscorpiidae

(Gervais, 1844)

Scorpio granosus Gervais 1844a: 233; 1844b: 65.
Chactas granosus : Karsch 1879: 111.
Megacormus granosus : Karsch 1881: 17; Kraepelin 1894: 151; 1899: 162; Pocock 1900: 417; 1902: 18: Borelli 1909: 224; Werner 1935: 285; Hoffmann 1938: 317; Stahnke 1973: 113; Díaz Nájera 1975: 4.

##### Type material.

**MEXICO:** Holotype female, depository unknown (Sissom 2000).

##### Diagnosis.

*Megacormus granosus* is most similar to *Megacormus segmentatus* by sharing pedipalp chela fingers margin straight lacking proximal notch and median lobe; nineteen trichobothria on patella retrolateral and seven on ventral surface. It can be separated from *Megacormus segmentatus* by having entirely densely granular surfaces on the femur, patella, tibia dorsal surfaces, prolateral leg surfaces, carapace, and tergites (Figures 1, 2), instead of having scattered fine granulation. *Megacormus granosus* is distinguished from *Megacormus gertschi*, *Megacormus grubbsi*, and *Megacormus segmentatus* by having the marginal and median lamella indistinguishable in females and vestigial in males, instead of having median lamella furrow in both sexes deep, completely separating marginal from median lamella. Males of *Megacormus granosus* have pedipalp chela fingers margin straight, but the male pedipalp fingers margin of *Megacormus gertschi* and *Megacormus grubbsi* are emarginated, bearing a proximal notch and median lobe that creates a gap when fingers close. *Megacormus granosus* has three trichobothria in series *et*, *em*, and six in *v*, whereas *Megacormus gertschi* has four, five and eight in *et*, *em* and *v* respectively; and *Megacormus grubbsi* has four in *em*.

##### Redescription.

The following redescription supplements Soleglad’s (1976) description and is based on 10 adult males, 3 adult females, 2 subadult females, 1 subadult male, 8 juvenile females and 4 juvenile males. Character variation is reported for the sexes as noted.

*Color and infuscation*: Base color yellowish to orange. Carapace: tergites, prolateral surface of legs, sternum, genital operculum, pectinal basal piece, fused lamella, metasoma, and telson, with dense, marbled infuscation (Figures 3–6). Chelicerae: manus base color yellowish with reticulated longitudinal infuscation, fingers moderately infuscate proximally. Pedipalps: base color orange with fuscous markings, all carinae densely infuscate. All trichobothrial bases with a bright yellowish areola. Legs: retrolateral surface yellowish. Spiracles light beige. Pectinal teeth whitish to light beige. Sternite III median and submedian surface densely infuscate, sternite IV–VII median carina infuscate, other surface immaculate. Telson vesicle ventral surface with three broad bands of infuscation flanking two submedian bands of yellowish base color, all surface infuscate dorsally. Aculeus base faintly infuscate, reddish distally.

**Figure 7, 8.**
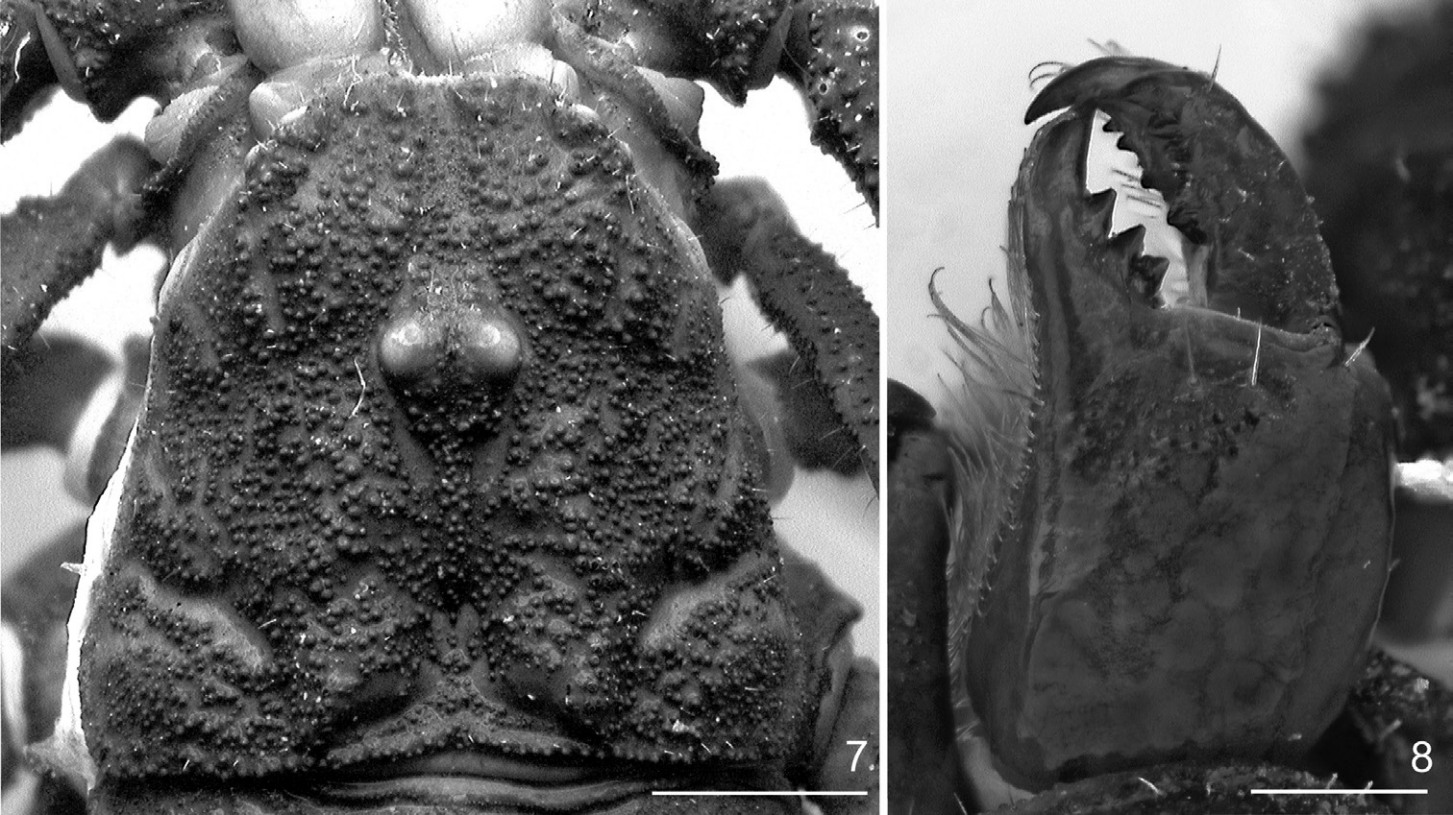
*Megacormus granosus* (Gervais, 1844). **7** dextral chelicera, dorsal view **8** dorsal carapace. Scale bar: 0.5 mm (**7**), 1 mm (**8**).

*Chelicerae*: Manus dorsal surface smooth, lustrous, with three macrosetae distally, decreasing in size from median to lateral surface. Movable finger, retrolateral margin with subdistal and medial denticles triangular, subequal; distal and basal denticle slightly larger; prolateral margin with three smaller, triangular, subequal denticles, situated in distal half of the finger; retrolateral distal finger size half of prolateral distal finger. Fixed finger margin with three denticles, proximal two adjacent and distal separate; distal denticle elongate and sharp (Figure 7); ventral surface of manus, fixed and movable finger with an interspaced tuff of setae with curved tips. Serrula absent.

*Carapace*: Length equal to 0.9 times the posterior width. Surface shagreened, with enlarge scattered granules covering entire surfaces (Figure 8). Distal margin with two pairs of macrosetae; emarginated, bilobed, with a shallow median notch, with extreme lateral sides curving placing lateral ocelli in a laterofrontal position. Two pairs of lateral ocelli of equal size, lateral ocular carinae strong, costate-granular. Median ocular tubercle raised, situated in anterior half of carapace. Superciliary carinae strongly (♀) or weakly (♂) granular, lower than median ocelli. Anteromedian sulcus deep and broad, with scattered granules; posteromedian, proximal half with a granular carina and distal half with a deep and broad depression; anterolateral, deep and narrow; posterior transverse shallow.

*Coxosternal region*: Sternum pentagonal, subequilateral, length equal to 0.8 times the width, with five to ten pairs of microsetae. Median sulcus of sternum with anterior and posterior margins broadened, moderately deep (♂), or very deep (♀). Coxa IV two and half times longer than coxa II. Coxae I–IV surfaces with scattered granules and margins densely granular; coxa II, prolateral subproximal margin with three oblique slit-like structures, adjacent to a moderate (♀) or low (♂) granular protuberance; coxae II–IV, prolateral carinae strongly granular (Figures 20, 21).

**Figures 9–17.**
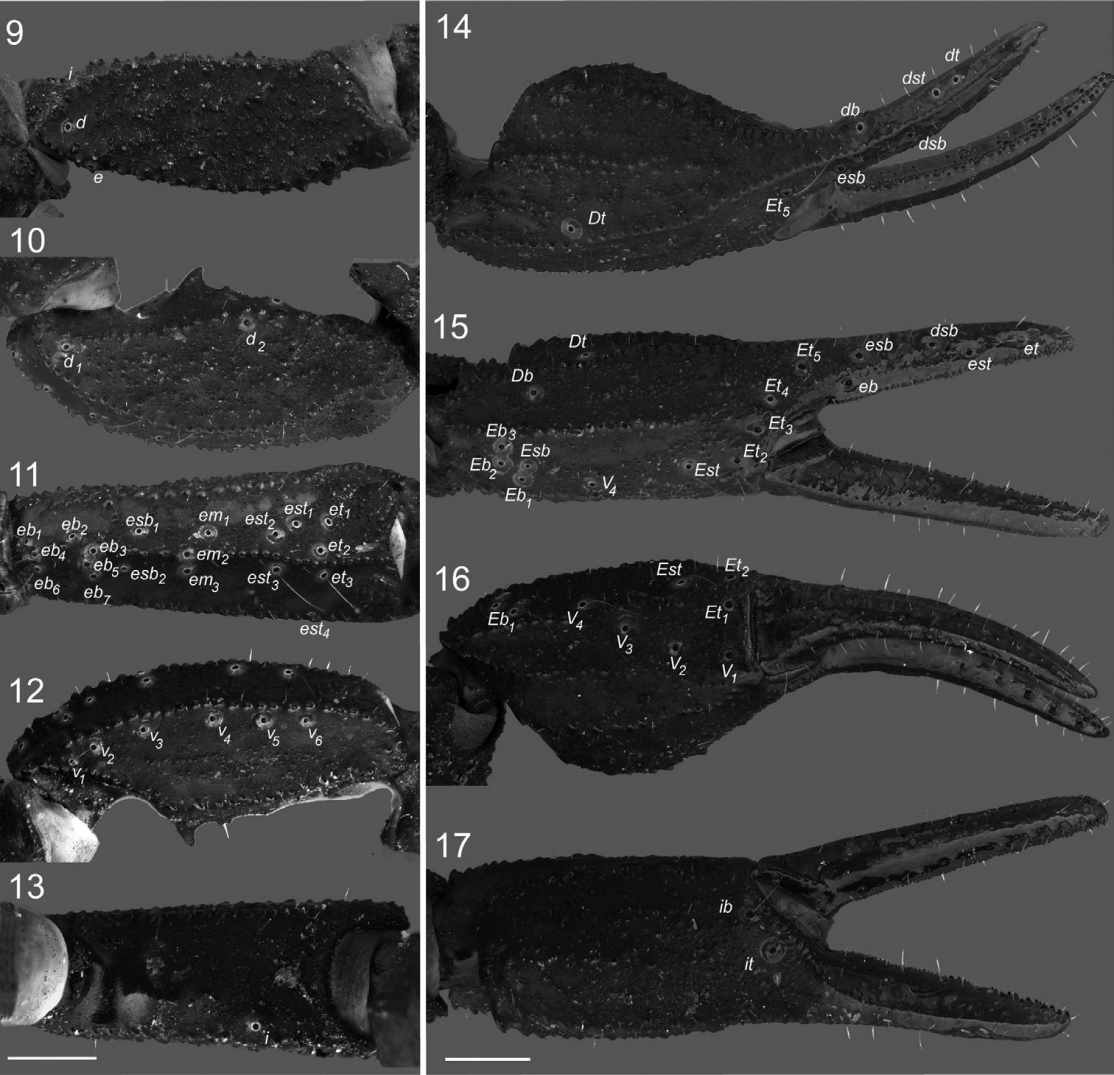
*Megacormus granosus* (Gervais, 1844). **9** dextral femur, dorsal view **10–13** dextral patella, dorsal, retrolateral, ventral and prolateral views **14–17** dextral chela, dorsal, retrolateral, ventral and prolateral views. Scale bars 0.5 mm.

**Figures 18, 19.**
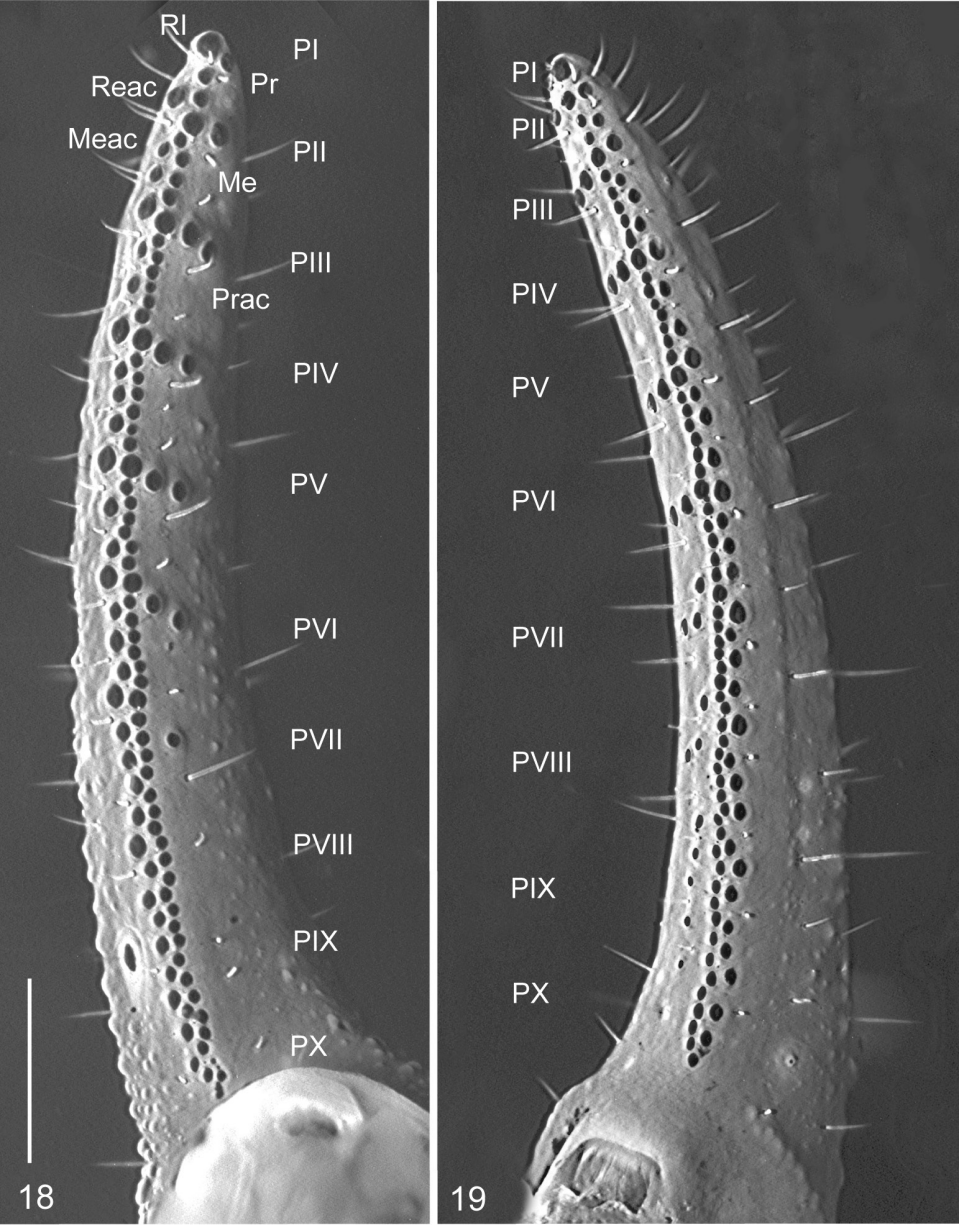
*Megacormus granosus* (Gervais, 1844). **18** dextral pedipalp movable finger **19** dextral pedipalp fixed fingers. Scale bars 0.5 mm.

*Pedipalps*, Femur prolateral, dorsal and retrolateral intercarinal surfaces shagreened (Figure 9), ventral surface with a cluster of fine granules medially. Dorsal prolateral, dorsal retrolateral, ventral prolateral and dorsal prolateral carinae complete, irregularly granular; retrolateral dorsosubmedian complete, weak proximally, becoming strongly granular distally; retrolateral ventral and ventral median carinae vestigial, reduced to few granules proximally; ventral retrosubmedian partial, with a scattered enlarged granules on proximal half; prolateral ventral vestigial, one or two median granules; prolateral ventrosubmedian partial, with enlarged granules on proximal fifth. Patella width 1.5 times greater than femur width. Dorsal intercarinal surfaces shagreened, prolateral, retrolateral, and dorsal sparsely finely granular. Dorsal prolateral, dorsal retrolateral, ventral prolateral, ventral retrosubmedian, and retrolateral median complete, granular; retrolateral dorsosubmedian absent; prolateral process reduced, expressed as a spiniform enlarged tubercle, prolateral median carina vestigial, expressed by one or two median granules (Figures 10–13). Chela length 1.9 times greater than femur and patella, width 1.6 times greater than patella and 1.1 than femur. Dorsal intercarinal surfaces shagreened, a dense field of minute and coarse granules subdistally, other surfaces with scattered minute and coarse granules. Dorsal retrolateral carina complete, strongly granular, extending to proximal four-fifths of the fixed finger, becoming weaker and smooth distally; dorsal retrosubmedian accessory vestigial, irregularly granular, restricted to trichobothrium *Dt*; dorsal median and dorsal retrosubmedian with an enlarged proximal tubercle, complete, irregular, granular forming two rows proximally, converging into a single row distally; prolateral dorsal, dorsal prosubmedian and dorsal prolateral, fused, irregular, with five to seven scattered granules proximally, minute and coarse granules medially, and coarse granules extending to fixed finger to the extent of trichobothrium *dsb*; retrolateral dorsal partial, minute granules on median two quarters; retrolateral dorsosubmedian vestigial, restricted to distal short row of coarse granules between trichobothria *Et_4_* and *Et_5_*; retrolateral median complete, strongly granular, ending at the level of trichobothria *Et_3_* and *Et_4_*; retrolateral subventral accessory and retrolateral subventral vestigial, restricted to a distal short row of coarse granules converging to trichobothrium *Et_2_*, commonly merging to ventral retrolateral carinae; retrolateral ventral partial, irregular, with minute granules restricted by trichobothria *Esb* and *Est*; ventral retrolateral complete, strongly granular, in some specimens forming a ring of granules around trichobothrium *V_4_*; ventral median partial, strongly granular proximally, becoming weak medially and merging with a field of granules distally, ventral retrolateral and ventral median forming an acute angle proximally, becoming parallel medially to distally; ventral prolateral and prolateral ventral complete, merging to a low tubercle proximally, multiple rows of granules curving to prolateral condyle distally; prolateral ventral accessory partial, restricted to midpoint of the manus as a multiple row of minute and coarse granules, prolateral median partial, irregular, coarse granules row restricted to proximal half (Figures 14–17). Pedipalp fixed and movable fingers: notches, lobes, and gap when fingers closed absent; dentate margin sublinear, compound, with multiple rows of prolateral, median and retrolateral denticles; prolateral, prolateral accessory, retrolateral and median denticles aligned in an oblique row angling retrolaterally in position III–VI. Fixed finger median row comprising six or seven denticle subrows with, commonly two, occasionally one denticle in position I, four to six in position II–VII; flanked by a two- or three-denticle retrolateral accessory median subrow, absent in position I; median subrows divided by six or seven retrolateral denticles, indistinguishable from median subrows’ denticles in position VII/VIII–X; median accessory subrows divided by five or six subpaired retrolateral accessory denticles, absent on I and undistinguishable from median accessory denticles subrows on VII/VIII–X; flanked by six or seven prolateral denticles and a subpaired prolateral accessory denticles in position III–VI, absent on I and II, vestigial to absent on VII–X. Movable finger median denticle row comprising six or seven median denticle subrows, zero to one in position I, three to seven in positions II–IX, zero on X; flanked by a two- or three-denticle median accessory denticles subrows, absent in position I; median subrows divided by seven to eight retrolateral denticles, indistinguishable in position IX and X, and median accessory subrows divided by six to seven subparied retrolateral accessory denticles, absent in position I, undistinguishable in position VII–X; flanked by nine prolateral denticles, position III–X subparied by prolateral accessory denticles, lower and less defined in positions VIII–X (Figures 18, 19).

*Trichobothrial pattern* Type C, neobothriotaxic. Femur trichobothria *d*, *e*, and *i* positioned proximally, equidistant; *d* on dorsal surface, *e* on ventral prolateral carina, *i* ventral to dorsal prolateral carina (Figure 9). Patella trichobothria *d_1_* and *d_2_* on dorsal surface proximal and medial respectively; *i* on prolateral distal half, ventral to dorsal prolateral carina; *eb_1_*–*eb_4_*, and *eb_5_*–*eb_6_* dorsal to retrolateral median carina; *esb_1_*, *em_1_*, *est_1_*, *et_1_*, *et_2_* and *eb_5_*–*eb_7_*, *esb_2_*, *em_3_*, *est_3_*, *et_3_* dorsal and ventral to retrolateral median carina respectively, *esb_2_*, petite, *em_2_* on retrolateral median carina, *v_1_*–*v_6_* proximal to ventral retrosubmedian carina (Figures 10–13). Variation in trichobothrial counts as follow: series *v*, 2 specimens, 5 left/5 right; 14, 6/6; 1, 6/5; 1, 7/7; *et*, 16, 3/3; 1, 3/2; *est*, 13, 4/4; 2, 3/4; 1, 3/3; *em*, 15, 3/3; 1, 3/2; *esb*, 15, 2/2; 1, 2/1. Chela trichobothrium *Db* on retrolateral surface between dorsal retrolateral and retrolateral dorsal carinae; *Dt* on dorsal surface at distal end of dorsal retrosubmedian accessory carina; series *db*–*dt* on dorsal and *db* on retrolateral surface, between denticle positions VIII and IX; series *eb*–*et* on retrolateral surface, *eb* between position VIII and IX; *esb* between positions VI and VII; *est* between positions V and VI, *et* at position IV; *Eb_1_*–*Eb_3_* on retrolateral surface, between retrolateral median and ventral retrolateral carinae; *Esb* petite, proximal to *Eb_1_*; *Et_1_* on dorsal surface close to retrolateral condyle, *Et_2_*–*Et_5_* on retrolateral surface, *Eb_2_*–*Eb_4_* on distal margin of manus, *Eb_4_* not petite, *Eb_5_* on base of movable finger; *V_1_*–*V_3_* on dorsal surface, equidistant, *V_4_* on ventral retrolateral carina; *ib* and *it* on distal margin of fixed finger (Figures 14–17).

*Legs*: Basitarsi, prolateral ventral and retrolateral ventral spinule rows partial, distal half with two or three sparse spinules on legs I–III, absent on leg IV; retrolateral and retrolateral dorsal rows absent on I–IV; macrosetal counts on legs I–IV, respectively: dorsal 2:2:2:2, retrolateral dorsal, 2:2:3:3; retrolateral ventral, 5:5:5:5; prolateral ventral, 4:4:4:4, all macrosetae not pigmented, translucent and shaped as tines; dorsal and retrolateral dorsal macrosetae arranged in two separate parallel rows on legs I–IV. Telotarsi I–IV, each with single irregular ventromedian row of scattered spinules and one ventrodistal spinule, flanked by prolateral and retrolateral rows of six macrosetae. Ungues short and curved.

**Figures 20–25.**
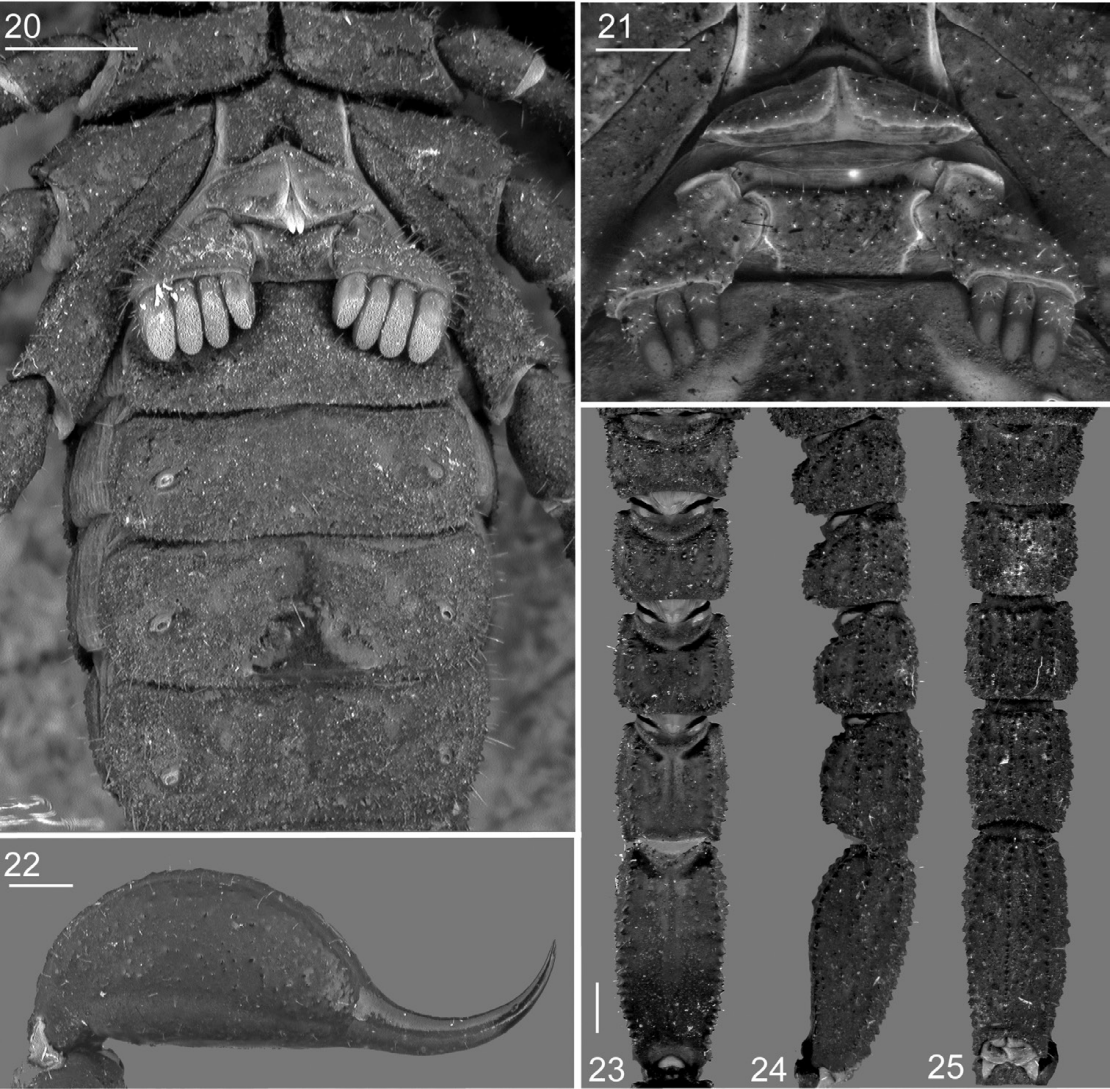
*Megacormus granosus* (Gervais, 1844). **20** ♂ dorsal coxosternal region, genital operculum, pectines, and sternites III–VI **21** ♀ pectines and genital operculum **22** telson lateral view **23–25** metasomal segments I–V dorsal, lateral, and ventral view. Scale bars: 0.5 mm, except Figure **21:** 0.25 mm.

*Genital operculum*: Wider than long, with four (♂) or six (♀) pairs of short and translucent macrosetae; sclerites free longitudinally, anterior margin fused on distal two thirds (♂) or fused longitudinally by a loose pleura folding into a valve covering the genital opening (♀). Genital papillae present, protruding posteriorly (♂) or absent (♀) (Figures 20, 21).

**Figure 26–28.**
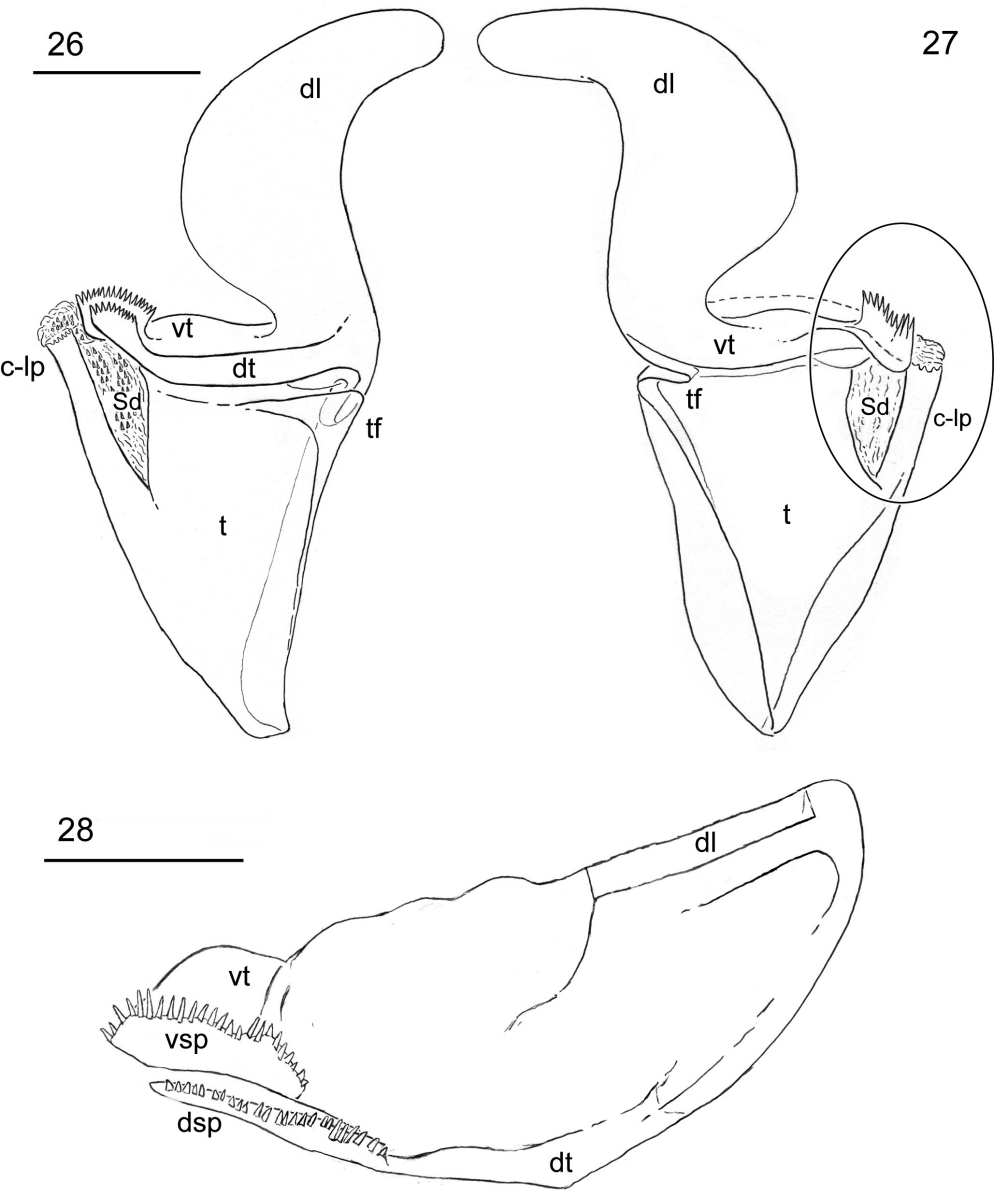
*Megacormus granosus* (Gervais, 1844). **26** ♂ dextral hemispermatophore dorsal view **27** hemispermatophore ventral view **28** transverse plate of dorsal and ventral troughs cross section view. Scale bars: 0.5 mm (**26, 27**); 0.2 mm (**28**).

*Hemispermatophore*: Distal lamina 1.1 times the length of trunk; tapering distally, basal constriction well-developed (Figures 26–28). Capsule’s dorsal and ventral troughs strongly sclerotized, merging into a complete, thick, transverse plate, dividing lamina and trunk (Figure 28). Marginal terminus of dorsal and ventral troughs with a spiculate processes with 25 and 24 irregular spines [in part Sissom’s (1994) accessory lobes], respectively. Hemi-mating plug gelatinous. Sperm duct formed by a spicule-coated membrane (*sensu* Jacob et al. 2004) connected to the spiculate processes of the dorsal and ventral troughs and to the crown-like process (*sensu* Jacob et al. 2004). Trunk broad proximally, tapering distally; crown-like process relatively long, with row of six to eight irregular spinules on the margin; truncal flexure and dorsal axial carinae well-developed (Figures 26–28).

*Pectines*: Basal piece with three or five pairs of macrosetae, proximal surface granular, V-shaped (♂) or isosceles trapezoidal (♀). Marginal and median lamellae nearly fused into one piece with a fine, shallow furrow (♂), or completely fused, indistinguishable furrow (♀). Fulcra absent. Pectinal teeth: three to four (♂) or one to four (♀). Pectines relatively short, fused lamella aligned with midpoint of coxa IV (Figures 20, 21).

*Tergites*: I–VI, intercarinal surfaces shagreened, densely covered with minute and coarse granules, posterior margin with rows of irregular granules (Figures 1 and 2); dorsal median and dorsal lateral carinae partial, costate-granular, restricted to proximal half (♂) or vestigial (♀). VII, intercarinal surfaces shagreened; dorsal median carina partial, restricted to anterior half of segment, costate-granular; dorsal sublateral carinae vestigial, comprising few serrate granules restricted to anterior half of segment; dorsal lateral and lateral median carinae converging anteriorly, serrate, posterior granules enlarged.

*Sternites*: Sternite III, surface around pectines shagreened; sternites IV–VI, surfaces smooth to weakly granular medially, shagreened laterally; spiracles minute, ovoid, 2.0 times longer than wide; sternite V, ventral surface distinct hyaline glandular area posteromedially, densely cover with micropores (♂) or absent (♀) (Figure 20). Sternite VII, intercarinal surfaces shagreened, without hyaline glandular area posteromedially; carinae obsolete, except ventral median, variable from a row of weak granules to coarse granules medially; ventrolateral with a row of coarse granules medially.

*Metasoma*: Length 0.9 times greater than mesosomal length (Table 1); segment I, 0.6; II, 0.7; III, 0.8; IV, 1.2; and V, 2.1 length greater than width; segment V width 0.9 times greater than telson width. Segments I–V, dorsal intercarinal surfaces scattered, finely granular, lateral and ventral shagreened. Dorsal lateral carinae complete, strongly serrate; lateral median carinae complete, serrate on I–III, weakly lobate posteriorly on IV, partial, reduced to anterior half, irregular scattered granules on V. Lateral inframedian carinae complete, with scattered, coarse granules on I, partial, granular, restricted to posterior half on II and III, vestigial and restricted to a posterior marginal tubercle on IV, absent on V. Ventral lateral carinae complete, serrate. Ventral submedian carinae vestigial, restricted to a marginal paired tubercle posteriorly on I–IV, absent on V. Ventral median carina complete, strongly serrate on I–V (Figures 23–25). Macrosetal counts on carinae of segments I–V, respectively: dorsal lateral, 4:4:4:5:7; lateral median, 4:4:5:5:3; lateral inframedian, 4:2:2:1:0; ventral lateral, 3:3:3:3:2; ventral sublateral, 0:0:0:0:1; ventral submedian, 3:3:3:3:4.

**Table 1.** Measurements (mm) of six adult males and three females of *Megacormus granosus* (Gervais, 1844).

		♂/♂/♂/♂/♂/♂/♀/♀/♀
Carapace	length	3.40/3.25/3.20/3.00/3.25/3.15/4.20/4.30/3.90
Anterior	width	2.00/1.80/1.80/1.60/1.80/1.75/2.40/2.50/2.35
Posterior	width	3.60/3.35/3.35/3.20/3.35/3.25/4.40/4.20/4.25
Femul	length	2.70/2.65/2.70/2.45/2.60/2.55/3.30/3.60/3.05
	width	1.10/1.00/0.95/0.90/1.00/1.00/1.30/1.30/1.30
	height	2.80/2.80/2.60/2.60/2.85/2.65/3.50/3.80/3.35
Patella	width	1.40/1.25/1.40/1.30/1.40/1.30/1.90/1.80/1.80
Chela	length	5.30/5.20/5.05/4.70/5.15/5.00/6.50/7.10/6.4
Manus	width	1.70/1.60/1.55/1.50/1.55/1.50/2.20/2.30/1.95
	height	1.40/1.20/1.35/1.05/1.45/1.15/1.70/1.70/1.75
Fixed finger	length	2.30/2.30/2.30/2.15/2.20/2.15/3.00/3.00/2.80
Movable Finger	length	3.00/2.95/3.00/2.70/2.85/2.85/3.90/4.10/3.75
Coxa II	length	1.30/1.35/1.25/1.20/1.40/1.25/1.50/1.70/1.75
Coxa IV	length	2.70/2.55/2.60/2.45/2.55/2.40/3.40/3.60/3.40
Sternum	length	0.80/0.70/0.70/0.65/0.75/0.85/1.00/0.90/1.05
Sternum	width	0.90/0.90/0.90/0.90/0.85/0.70/1.00/0.70/1.15
Mesosoma	length	5.20/4.70/5.35/4.35/5.30/4.90/7.00/7.70/6.80
Metasoma	length	8.50/4.48/4.60/4.23/4.30/4.10/8.60/9.10/4.25
Segment I	length	0.90/1.00/1.10/1.10/1.05/1.00/1.00/1.20/1.05
	width	2.00/1.95/1.95/1.65/1.95/1.80/2.10/2.20/2.10
	height	1.70/1.50/1.60/1.50/1.55/1.60/1.70/1.70/1.85
Segment II	length	1.20/1.20/1.25/1.25/1.15/1.10/1.20/1.20/1.30
	width	1.90/1.85/1.85/1.60/1.75/1.70/1.90/2.00/1.90
	height	1.70/1.35/1.55/1.40/1.35/1.45/1.50/1.70/1.65
Segment III	length	1.40/1.35/1.40/1.45/1.30/1.20/1.30/1.30/1.30
	width	1.80/1.75/1.75/1.60/1.70/1.65/1.80/1.90/1.80
	height	1.60/1.30/1.55/1.40/1.40/1.35/1.50/1.60/1.65
Segment IV	length	1.80/1.90/1.95/1.65/1.80/1.80/1.90/1.90/1.75
	width	1.70/1.60/1.65/1.50/1.55/1.50/1.60/1.70/1.60
	height	1.70/1.40/1.55/1.40/1.50/1.35/1.50/1.60/1.60
Segment V	length	3.20/3.50/3.50/3.00/3.30/3.10/3.20/3.50/3.10
	width	1.50/1.60/1.65/1.45/1.55/1.45/1.50/1.50/1.55
	height	1.60/1.25/1.50/1.35/1.45/1.25/1.40/1.50/1.55
Telson	length	4.20/3.90/4.25/3.80/4.00/3.80/4.50/4.50/4.30
Vesicle	length	2.90/2.25/2.45/2.30/2.35/2.20/2.90/2.60/2.30
	width	1.90/1.65/1.65/1.55/1.70/1.50/1.50/1.60/1.50
	height	1.30/1.30/1.45/1.35/1.35/1.30/1.30/1.30/1.25
Aculeus	length	1.30/1.65/1.80/1.50/1.65/1.60/1.60/1.90/2.00
Total	length	21.30/16.33/17.40/15.38/16.85/15.95/24.30/25.60/19.25

*Telson*: Vesicle globose, length 1.4 times greater than width (Table 1); dorsal surface with finely punctuated and scattered minute granules (♂) or smooth (♀); ventral surface scattered with minute and coarse granules, carinae obsolete, with four or five pairs of short translucent macrosetae, annular ring moderately developed (Figure 22). Aculeus, fairly elongated, laterobasal microserration and subaculear tubercle absent, venom delivery openings slit-like, paired.

##### Distribution.

*Megacormus granosus* has been reported in the vicinities of the National Park Pico de Orizaba, on the slopes of the Trans-Mexican Volcanic Belt facing the Gulf of Mexico, and between Orizaba and Huatusco, Veracruz.

##### Ecology.

All adult males were collected by pitfall traps, suggesting high motility within the leaf litter. They were particularly abundant in the May 2012 expedition. This behavior in males and the period of the year may be related to the mating season of the species. All adult females and immatures of both sexes were collected exclusively using Berlese funnels, suggesting these are comparatively less mobile. A total of 72 Berleses and 180 pitfalls were used to sample two hectares, of which 18 (25%) and 11 (5%), caught 18 and 9 specimens respectively. These yields are consistent with low population density of this species; adult males are particularly rare. The habitat of, and behavior exhibited by, this species as well as its cryptic morphology (color resembling substrate; relative small size) are congruent with a humiculous ecomorphotype (Prendini 2001).

##### Remarks.

The catalog of the scorpions of the world (Fet et al. 2000) indicates that the location of *Megacormus granosus* type material is either unknown or lost. It is important to investigate the whereabouts of Gervais’ unique specimen to verify the holotype, or failing this, to designate a neotype. Workable keys to the species of *Megacormus* are provided in Sissom (1994). The genus *Megacormus* is under revision by O.F. Francke (per. comm.).

## Discussion

The illustrations of the *Megacormus granosus* hemispermatophore presented in Figures 26 and 27 are congruent with that of Stockwell (1989: p. 381, figure 217), but differ from that of Sissom (1994: p. 269, figures 8–10), who illustrate accessory lobes associated to the sperm duct (Sissom’s *acc*, figure 8). According to our findings, the accessory lobes are the termini of both the dorsal and ventral trough margins with a spiculate process (Figures 26, 27) and are not independent lobes as suggested by Sissom’s illustration (Figure 28). Furthermore, the basic conformation of the sperm duct, with the spicule-coated membrane and the crown-like process, appears to be uniform in these species. Although intra- and inter-specific comparative work is needed, we hypothesize that the capsular region of the hemispermatophore of *Megacormus* might carry little information to diagnose species of the genus, as demonstrated in other species complexes (Jacob et al. 2014, Santibañez-López and Francke 2010) and predicted by other studies (Song and Bucheli 2010, Peretti 2010).

## Supplementary Material

XML Treatment for Megacormus granosus

## References

[B1] Alvarez-Padilla F Hormiga G (2007) A protocol for digesting soft tissue and mounting spiders for scanning electron microscopy. Journal of Arachnology 35: 538–542. doi: 10.1636/Sh06-55.1

[B2] Birula A (1917) Arachnoidea Arthrogastra Caucasica. Pars I. Scorpiones. Zapiski Kavkazskogo Muzeya [Mémoires du Musée du Caucase], Imprimerie de la Chancellerie du Comité pour la Transcaucasie, Tiflis A (5): 253 pp [In Russian. English translation: Byalynitskii-Birulya, A.A. 1964. Arthrogastric Arachnids of Caucasia. 1. Scorpions. Israel Program for Scientific Translations, Jerusalem, 170 pp.]

[B3] Borelli A (1909) Scorpioni raccolti dal Prof. F. Silvestri. Bollettino del Laboratorio di zoologia generale e agraria della R. Scuola Superiore d’Agricoltora in Portici 3: 222–227.

[B4] Coddington JA (1983) A temporary slide mount allowing precise manipulation of small structures. In: Kraus O (Ed.) Taxonomy, biology and ecology of Araneae and Myriapoda. Verhandlungen des Naturwissenschaftlichen Vereins in Hamburg 26: 291–292.

[B5] Díaz Nájera S (1975) Lista y datos de distribución geográfica de los alacranes de México (Scorpionida). Revista de Investigación de Salud Pública 3: 263–276. 1179075

[B6] Francke OF (1979) Observations on the reproductive biology and life history of *Megacormus gertschi* Diaz (Scorpiones: Chactidae; Megacorminae). Journal of Arachnology 7: 223–230.

[B7] Hoffmann CC (1931) Monografías para la entomología médica de México. Monografía Num. 2, Los escorpiones de México. Primera parte: Diplocentridae, Chactidae, Vaejovidae. Anales del Instituto de Biología Universidad Nacional Autónoma de México 2: 291–408.

[B8] Hoffmann CC (1938) Nuevas consideraciones acerca de los alacranes de México. Anales del Instituto de Biología Universidad Nacional Autónoma de México 9: 318–337.

[B9] Fet V Sissom WD Lowe G Braunwalder ME (Eds) (2000) Catalog of the Scorpions of the World (1758–1998). The New York Entomological Society, New York, 690 pp.

[B10] Gervais P (1844a) Un nouveau scorpion. Archives Museum Histoire Naturalle Paris 4: 23–234

[B11] Gervais P (1844b) Scorpions. In: Alkenaer CA Gervais PM (Eds) Histoire naturelle des Insects Apteres. Librario Encyclop Roret, Paris, 14–74.

[B12] González-Santillán E Prendini L (2013) Redefinition and generic revision of the North American vaejovid scorpion subfamily Syntropinae Kraepelin, 1905, with descriptions of six new genera. Bulletin of the American Museum of Natural History 384: 1–71. doi: 10.1206/830.1

[B13] González-Santillán E Prendini L (2014) Phylogeny of the North American Vaejovid Scorpion Subfamily Syntropinae Kraepelin, 1905, based on morphology, mitochondrial and nuclear DNA. Cladistics: 1–65. doi: 10.1111/cla.12091 10.1111/cla.12091 34772267

[B14] Jacob A Gantenbein B Braunwalder ME Nentwig W Kropf C (2004) Complex male genitalia (hemispermatophores) are not diagnostic for cryptic species in the genus *Euscorpius* (Scorpiones: Euscorpiidae). Organism, Diversity and Evolution 4: 59–72. doi: 10.1016/j.ode.2003.11.002

[B15] Karsch F (1889) Scorpionologische Beiträge. I. Mitteilungen der Munchener Entomologischen Verein 3: 6–22.

[B16] Karsch F (1881) Ueber eine neune Gattung Skorpione. Archiv für Naturgeschichte, Berlin 57: 16–18.

[B17] Kraepelin K (1894) Revision der Skorpione. II Scorpionidae und Bothriuridae. Jahrbuch der Hamburgischen Wissenschaftlichen Anstalten 11: 1–248.

[B18] Kraepelin K (1899) Skorpiones und Pedipalpi. In: Schultz FE (Ed.) Das Tierreich. Friedlander, Berlin, 1–265.

[B19] Lamoral B (1979) The scorpions of Namibia. Annals of the Natural Museum 23: 497–784.

[B20] Lourenço WR Sissom WD (2000) Scorpiones In: Llorente B González E Papavero N (Eds) Biodiversidad, taxonomía y biogeografía de artrópodos de México: Hacia una síntesis de su conocimiento. UNAM, México, 115–135.

[B21] Mattoni CI Ochoa JA Ojanguren Afilastro AA Prendini L (2011) *Orobothriurus* (Scorpiones: Bothriuridae) phylogeny, Andean biogeography, and the relative importance of genitalic and somatic characters. Zoologica Scripta 41: 160–176. doi: 10.1111/j.1463-6409.2011.00508.x

[B22] Peretti AV (2010) An ancient indirect sex model: single and mixed patterns in the evolution of scorpion genitalia. In: Leonard JL Córdoba-Aguilar A (Eds) The evolution of primary sexual characters in animals. Oxford University Press, Oxford.

[B23] Pocock RI (1900) Some new or little-known Neotropical scorpions in the British Musuem. The Annals and Magazine of Natural History 5: 469–478. doi: 10.1080/00222930008678315

[B24] Pocock RI (1902) Arachnida, Scorpiones, Pedipalpi and Solifugae. Biologia Centrali-Americana. Taylor and Francis, London, 71 pp.

[B25] Prendini L (2001) Substratum specialization and speciation in southern African scorpions: the effect hypothesis revised. In: Fet V Selden PA (Eds) Scorpions 2001. In: Memoriam Gary Polis. Burham Beeches, Burks, British Arachnological Society, 113–138.

[B26] Santibañez-López CE Francke OF (2013) Redescription of *Diplocentrus zacatecanus* (Scorpiones: Diplocentridae) and limitations of the hemispermatophore as a diagnostic trait for genus *Diplocentrus*. Journal of Arachnology 41: 1–10. doi: 10.1636/Ha12-65.1

[B27] Sissom WD (1994) Systematic studies on the genus *Megacormus* (Scorpiones, Chactidae, Megacorminae), with descriptions of a new species from Oaxaca, Mexico and of the male of *Megacormus segmentatus* Pocock. Insecta Mundi 8: 265–272.

[B28] Sissom WD (2000) Family Vaejovidae. In: Fet V Sissom WD Lowe G Braunwalder ME (Eds) Catalog of the Scorpions of the World (1758–1998). The New York Entomological Society, New York, USA, 503–553.

[B29] Soleglad ME (1976) A revision of the scorpion subfamily Megacorminae (Scorpionida: Chactidae). The Wasmann Journal of Biology 34: 251–303.

[B30] Soleglad ME Sissom WD (2001) Phylogeny of the family Euscorpiidae: a major revision. In: Fet V Selden PA (Eds) Scorpions 2001. In: Memoriam Gary A. Polis. British Arachnological Society: Burnham Beeches, Buckinghamshire, UK, 25–111.

[B31] Song H Bucheli SR (2010) Comparison of phylogenetic signal between male genitalia and non-genitalia characters in insect systematics. Cladistics 26: 23–35. doi: 10.1111/j.1096-0031.2009.00273.x 10.1111/j.1096-0031.2009.00273.x 34875749

[B32] Stahnke HL (1970) Scorpion nomenclature and mensuration. Entomological News 81: 297–316. 5417256

[B33] Stahnke HL (1973) Revision and keys to the higher categories of Vaejovidae (Scorpionida). Journal of Arachnology 15: 107–141.

[B34] Stockwell SA (1989) Revision of the phylogeny and higher classification of scorpions (Chelicerata). PhD Thesis, University of California, Berkeley.

[B35] Werner F (1935) Scorpions und Pedipalpi. In: Bronn HG Klassen und Ordnungen des Terriechs, Leipzig 3: 1–316.

